# Assessment of factors associated with depression and anxiety among pwMS in Saudi Arabia

**DOI:** 10.1186/s12883-022-02632-2

**Published:** 2022-03-25

**Authors:** Safanah AlSaeed, Nuha M. Alkhawajah, Mohsen Ayyash, Salman Aljarallah, Rola Alarieh, Amani Abu-Shaheen

**Affiliations:** 1 King Fahad Medical City, Riyadh, Saudi Arabia; 2 College of Medicine, King Saud University, Riyadh, Saudi Arabia; 3 School of Mathematical Sciences, Universiti Sains Malaysia, USM, 11800 Penang, Malaysia; 4 Department of Nuerology, King Fahad Medical City, Riyadh, Saudi Arabia; 5 Research Center, King Fahad Medical City, Riyadh, Saudi Arabia

**Keywords:** Anxiety, Depression, Fatigue, Multiple sclerosis, KSA

## Abstract

**Background:**

Multiple sclerosis (MS) is an inflammatory chronic disease that is characterized by an increased prevalence of adverse mental health outcomes in patients with MS (pwMS). The main aim of this study is to investigate the factors of depression and anxiety in pwMS in the Kingdom of Saudi Arabia (KSA).

**Materials and methods:**

This is a cross-sectional study conducted in KSA during the period from March to June 2020. Participants were recruited from the Neuroimmunology clinics in King Fahad Medical City (KFMC) and King Saud University medical city (KSUMC)in Riyadh City, KSA. The Hospital Anxiety and Depression Scale (HADS) was used to measure depression and anxiety. Fatigue Severity Scale (FSS) was used to measure fatigue in pwMS. A simple random sampling technique was utilized to select participants and the data were analyzed using SPSS v.24.0.

**Results:**

A total of 529 participants participated in this study with a response rate of 53.1%. The prevalences of anxiety and depression were 35.3% and 19.7%, respectively. The findings also revealed that depression was more likely to be significantly affected by being male, low education, unemployment, physical inactivity, and fatigue but the anxiety was significantly affected by region, unemployment, short duration since last MS relapse, physical inactivity, and fatigue.

**Conclusion:**

Anxiety and depression are not uncommon in pwMS. Given their impact on the lives of affected patients, early detection and management of these symptoms and their associated factors are crucial.

## Introduction

MS is one of the debilitating and pathologically complex neurological disorders that affect the central nervous system [1]. Recent estimates of MS prevalence exhibited an increasing rate of disease from 2.3 million people in 2013 to 2.8 million in 2020 worldwide including patients who are under 18 years old [2]. In the Kingdom of Saudi Arabia (KSA), the total number of pwMS was 13,120 with a prevalence of 41 patients per 100,000 people [2]. AlJumah et al. found that the prevalence of MS was 7.7 per 100,000 people from a sample of pwMS (age range from 11 to 63 years) from 20 hospitals in KSA and the projected overall prevalence of MS was 40.4 per 100,000 people in 2017 [3]. In general, MS affects females more than males with about a 2:1 female to male ratio worldwide [4]. In KSA, a similar female to male ratio was also estimated [3].

MS course may differ among patients; as some may experience a relapsing and remitting course while others may have progressive disease [5]. Factors such as viral infections, lack of vitamin D, family or genetic history, mental health adverse outcomes (e.g., depression, anxiety, and stress), residence area, and lifestyle may play a vital role in raising the prevalence of developing MS disease [6].

Prior studies have shown that MS is associated with a significant psychological burden including anxiety, depression, and stress which in turn leads to negative effects on a patient's lifestyle [7–12]. The estimated prevalence of anxiety and depression in pwMS ranged from 14 to 41% and from 14 to 54%, respectively [7–11]. For example, a systematic review and meta-analysis study showed that the estimated pooled prevalence of depression was 30.5% and it was 22.1% for anxiety [8]. Different studies investigated the factors influencing the levels of depression and anxiety in pwMS [9, 12–15]. Karimi et al. showed that depression was significantly associated with a job, education, and economic status while anxiety was significantly associated with the economic status of pwMS [9]. A study by Pham et al. indicated that higher odds of anxiety were associated with higher depression, low education, and decreased quality of life [12]. However, Alsaadi et al. showed that factors including age, sex, education, disease duration, and Expanded Disability Status Scale (EDSS) scores were not significantly associated with anxiety and depression in the United Arab Emirates [13]. A study conducted in Iran showed that depression in pwMS can be significantly predicted by physical problems, body pain, and fatigue [14]. A study in KSA showed that the prevalence of depression symptoms in pwMS was significantly associated with employment status and smoking while the severity of depression was associated with the level of education [15].

On the other hand, it is important to note that there are some invisible neuropsychiatric symptoms in pwMS that do affect their quality of life (QoL) and physical activity including fatigue [7, 16–20], dysphagia [21, 22], sexual dysfunction [23, 24], and bladder/bowel dysfunctions [25–28] in addition to mood disorders [20], cognitive impairments [20, 29–32], pain [33, 34], and vision changes [20, 35]. A study by Altmann et al. indicated that sexual dysfunction is significantly predicted by EDSS but not by anxiety and depression [23]. Alvino et al. showed that bowel dysfunction was significantly higher in women and MS-associated disability measured by EDSS [25]. Recent studies showed that about 50% of pwMS experienced bowel dysfunction that can result in distress and humiliation [27, 28]. A recent review indicated that lower uniary tract symptoms in pwMS affect their QoL and a significant increased risk of mortality [36]. Previous studies also domnestrated that cognitive impairments (e.g., episodic memory decline, slowed cognitive processing speed, verbal fluency changes, difficulties in executive function, and reductions in visuospatial analysis) are prevalent among 70% of pwMS [29, 30], which are mostly presented at the time of disease diagnosis [31, 32]. pwMS also reported pain associated with MS including trigeminal neuralgia, neuropathic, trigeminal neuralgia, burning limb, and musculoskeletal pain that might affect their lives [33, 34]. Lakin et al. provided a narrative review for most of these symptoms [20].

Therefore, considering the increasing prevalence of MS in KSA, and its associated adverse psychological outcomes of depression and anxiety, there is a need to investigate the sociodemographic and clinical factors associated with these adverse outcomes. Hence, this study aims to investigate the factors influencing depression and anxiety in pwMS in KSA.

## Materials and methods

### Study design and settings

This is a cross-sectional study conducted in KSA during the period from March to June 2020. Participants were recruited from the neuroimmunology clinics in King Fahad Medical City (KFMC) and King Saud University Medical City (KSUMC) in Riyadh, KSA. We also recruited patients utilizing a database obtained from the Arfa MS association. Arfa MS association is a non-profit association supported by the Ministry of Human Resources and Social Development that aims to support patients and their families in many aspects by improving the QoL physically, psychologically as well as socially.

### Sample

The population of the current study is all patients diagnosed with MS from the three cohorts described above. The inclusion criteria were all pwMS who are 18 years and above, can read and write, have at least 6 months history of MS, have no walking difficulty (i.e., by Is it by asking respondents and by reviewing their medical records in their respective hospitals), having no history of relapse in the last two months, and having no other acute or chronic medical conditions such as cancer, cardiovascular, respiratory, or hepatic diseases. Therefore, the total eligible population size is 1700 patients. Establishing a power of 80% with a 2% margin of error and 95% confidence interval, the estimated sample size is 996 patients. Simple random sampling was used to select pwMS participants.

### Instrument and data collection

The instrument of the current study comprises three sections. The first section pertains to demographic information including participants’ age, sex, region, marital status, nationality, educational level, and work status in addition to clinical characteristics including duration of disease diagnosis, last MS relapse time, and daily physical activity. The second section measures fatigue using the Fatigue Severity Scale (FSS). FSS consists of nine statements describing the severity and impact of fatigue ranging from 1 (strongly disagree) to 7 (strongly agree) with possible total minimum and maximum scores of 9 and 63 points, respectively. Total FSS scores are usually reported as the mean score over the nine items, and higher scores indicate higher severity [37]. This study used the validated FSS Arabic version that demonstrated acceptable test-retest reliability, internal consistency, and psychometric properties [38]. The third section consists of Hospital Anxiety and Depression Scale (HADS) to measure anxiety and depression [39]. The scale includes 14 items assessing anxiety (7‑item) and depression (7‑item), which are rated from 0 to 3. The scores in each subscale are computed by summing the corresponding items, with minimum and maximum scores of 0 and 21, respectively for anxiety and depression separately. It can be categorized as follows: from 0 to 7 points is considered as normal, from 8 to 10 as a borderline case, and from 11 to 21 as a case (anxiety or depression) [39]. The present study utilized the validated Arabic version of HADS [40].

For data collection, an online version of the instrument was distributed to potential study participants. Before the questionnaire begins, the study objectives were explained to the patients, and were asked to agree to provide consent for participation in this study.

### Data analysis

The variables of this study were described in terms of mean $\pm$ SD (i.e., SD: standard deviation) and frequencies (percentages) as appropriate. Statistical differences in average scores of anxiety and depression according to categorical independent variables using the independent samples t-test or One-Way ANOVA as appropriate. Spearman’s correlation test was performed to assess the correlation between outcome variables and independent continuous variables. Furthermore, anxiety and depression scores were separately regressed on the independent variables by fitting multiple linear regression models for both outcomes. A two-tailed *p*-value of less than 0.05 was considered statistically significant. All statistical analyses were performed using the Statistical Package for Social Sciences (SPSS v.24.0; SPSS Inc., Chicago, IL, USA) package.

### Ethical considerations

The current study was reviewed and approved by the Institutional Review Board (IRB) of KSUMC reference (#20/0714) and KFMC (#21-412). An electronic informed consent was obtained before filling the questionnaires. Participation in this study was completely voluntary and not compulsory.

## Results

In this study, a total of 529 pwMS were studied with a response rate of 53.1% (i.e., 529/996). Of them, 216 (40.8%) were aged 30 to 39 years old. More than two-thirds were females (*n* = 355, 67.1%), with a female to male ratio of 2:1, and had a university education (*n* = 365, 69%). More than one-half of them were not married (*n* = 288, 54.4%) and unemployed (*n* = 289, 54.6%). Most respondents were residing in the central region of KSA (*n* = 321, 60.7%) and the majority of them were Saudis (*n* = 458, 86.6%). The average number of years after MS diagnosis was 7.1 (SD: 5.5) years with a minimum and maximum duration of diagnosis of 1 and 28 years. 241 (45.6%) participants had more than 18 months history since last MS relapse with an average of 28.5 (SD = 26.5) months and a range of 3 to 192 months. Furthermore, 213 (40.3%) participants had 58.0 (SD = 32.5) minutes of physical activity per day (Table 1). In addition, the average score of fatigue was 4.6 $\pm$ 1.8.

**Table 1:** Descriptive statistics of the study variables

Characteristics		N	%
Age groups (years)	18 -29	190	35.9
	30 -39	216	40.8
	40 – 49	105	19.8
	$\ge$ 50	18	3.4
Sex	Male	174	32.9
	Female	355	67.1
Marital status	Married	241	45.6
	Not married	288	54.4
Region	Central	321	60.7
	Eastern	73	13.8
	Western	73	13.8
	Northern	40	7.6
	Southern	22	4.2
Nationality	Saudi	458	86.6
	Non-Saudi	71	13.4
Education	Secondary or lower	104	19.7
	Diploma	60	11.3
	University	365	69.0
Work status	Employed	240	46.4
	Unemployed	289	54.6
Last MS relapse (in months)	3 – 6	126	23.8
	7 – 12	139	26.3
	13 – 18	23	4.3
	$>$ 18	241	45.6
Physical activity	Yes	213	40.3
	No	316	59.7

**Fig. 1:**
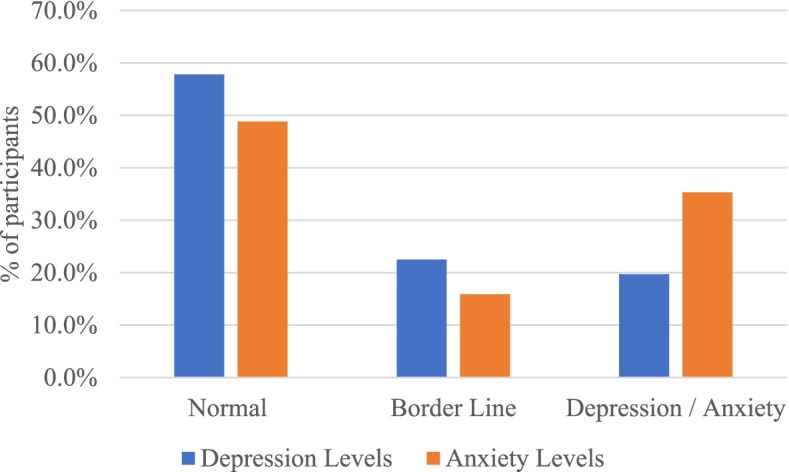
Distribution of depression and anxiety levels among pwMS

The results indicate that 104 (19.7%) pwMS had depression and 119 (22.5%) had a borderline level of depression (Fig. 1) with an overall mean score of 6.8 $\pm$ 4.3. The pwMS aged 50 years and above had the highest mean depression score (8.6 $\pm$ 4.7) and those with an age range from 18 to 29 years had the lowest mean depression score (6.5 $\pm$ 4.4), indicating that depression levels increase with an increase in patients’ age. However, differences according to age groups were not statistically significant (*p*-value = 0.113). The average scores of depression in males and females were 7.1 $\pm$ 4.3 and 6.6 $\pm$ 4.3 respectively, showing no significant statistical difference among them (*p*-value = 0.197). With respect to marital status, the average depression score in unmarried patients was (7.0 $\pm$ 4.3) higher than in married patients (6.4 $\pm$ 4.3), but this difference did not reach statistical significance (*p*-value = 0.102). As for nationality, there was no significant statistical difference in mean scores of depression levels among Saudis (6.7 $\pm$ 4.3) and non-Saudis (6.9 $\pm$ 4.2) (*p*-value = 0.653). Regarding region of residence, the highest mean score of depression was found in the southern region (8.1 $\pm$ 3.7) and the lowest was found in the northern region (6.4 $\pm$ 3.8), but with no significant statistical differences (*p*-value = 0.191).

Regarding educational levels, the highest and lowest mean depression levels were attached to the patients who had completed secondary or lower (8.4 $\pm$ 4.0) and diploma education (5.5 $\pm$ 4.2), respectively. In the meantime, the average depression score of patients who had completed university education was 6.5 $\pm$ 4.3. The results showed that differences in average scores of depression by education were statistically significant (*p*-value < 0.001). Although these differences were evident between patients with secondary or lower, and those who had either diploma (*p*-value < 0.001) or university education (*p*-value < 0.001), the difference between patients who had a diploma and university educational levels was not statistically significant (*p*-value = 0.202). As for work status, unemployed patients had a higher average score of depression (7.3 $\pm$ 4.4) in comparison to employed patients (6.1 $\pm$ 4.1) (*p*-value = 0.001). Concerning physical activity, patients without physical activity had a higher mean depression score (8.4 $\pm$ 3.8) than those with physical activity (5.7 $\pm$ 4.3) (*p*-value < 0.001) (Table 2). The findings also revealed a statistically significant direct association between the disease duration and depression ($\rho$ = 0.11, *p*-value = 016). Moreover, the time of the last MS relapse was inversely correlated with depression levels ($\rho$ = – 0.12, *p*-value = 0.006). That is a patient who had the shortest time since the last MS relapse had higher depression levels. Moreover, fatigue was significantly directly correlated with depression, ($\rho$ = 0.29, *p*-value < 0.001) (Table 3).

**Table 2:** Average scores of depression and anxiety by respondents’ demographics

Variables		Depression			Anxiety		
		Mean	SD	*p*-value	Mean	SD	*p*-value
Age	18 -29	6.5	4.4	0.113	8.7	5.1	0.539
	30 -39	6.6	4.2		8.1	5.2	
	40 – 49	7.3	4.2		8.6	5.7	
	$\ge$ 50	8.6	4.7		9.4	5.3	
Sex	Male	7.1	4.3	0.197	8.9	5.3	0.239
	Female	6.6	4.3		8.3	5.2	
Marital status	Married	6.4	4.3	0.102	8.2	5.1	0.306
	Not married	7.0	4.3		8.7	5.4	
Region	Central	6.5	4.3	0.191	8.1	5.0	< 0.001
	Eastern	6.9	4.9		7.5	5.9	
	Western	7.5	4.3		10.1	4.8	
	Northern	6.4	3.8		7.9	4.6	
	Southern	8.1	3.7		12.1	6.0	
Nationality	Saudi	6.7	4.3	0.653	8.5	5.3	0.978
	Non-Saudi	6.9	4.2		8.4	5.2	
Education	Secondary or lower	8.4	3.9	< 0.001	10.0	5.2	0.002
	Diploma	5.5	4.2		7.6	4.6	
	University	6.5	4.3		8.2	5.3	
Work status	Employed	6.1	4.1	0.001	7.7	5.1	0.002
	Unemployed	7.3	4.4		9.1	5.3	
Physical activity	Yes	5.7	4.3	< 0.001	7.4	4.9	< 0.001
	No	8.4	3.8		10.1	5.2	

- *p*-values for pairwise comparison of mean depression scores between educational levels: secondary or lower versus diploma: < 0.001; secondary or lower versus university: < 0.001; diploma versus university: 0.202

- *p*-values for pairwise comparison of mean anxiety scores between regions: central versus eastern: 0.914; central versus western: 0.064; central versus northern: 0.998; central versus southern: 0.017; eastern versus western: 0.047; eastern versus northern: 0.997; eastern versus southern: 0.010; western versus northern: 0.291; western versus southern: 0.657; northern versus southern: 0.051

- *p*-values for pairwise comparison of mean anxiety scores between educational levels: secondary or lower versus diploma: 0.017; secondary or lower versus university: 0.005; diploma versus university: 0.772

**Table 3:** Correlations between depression and anxiety with continuous independent variables

Variables		Disease duration	Last MS relapse	Fatigue
Depression	*ρ*^a^	0.11	- 0.12	0.29
	*p*-value	0.016	0.006	< 0.001
Anxiety	*ρ*^a^	0.002	- 0.15	0.19
	*p*-value	0.967	< 0.001	< 0.001

^a^Spearman correlation coefficient

The results also showed that 187 (35.3%) had anxiety and 84 (15.9%) had a borderline level of anxiety with a total mean score of 8.5 $\pm$ 5.2. The respondents in the age group of 50 years and above had the highest score of anxiety (9.4 $\pm$ 5.3) while those in the age group of 30 to 39 years had the lowest score of anxiety (8.1 $\pm$ 5.2). But the differences in anxiety average scores were not found to be statistically different by age groups (*p*-value = 0.539). Although males and unmarried patients had higher average scores, (8.9 $\pm$ 5.3) and (8.7 $\pm$ 5.4) respectively, the differences did not reach statistical significance. According to marital status, participants who were not married scored a higher mean score of anxiety (8.7 $\pm$ 5.4) than married ones (8.2 $\pm$ 5.0) indicating no statistically significant variation (*p*-value = 0.306). Furthermore, Saudi participants had a somewhat insignificantly higher average score of anxiety (8.5 $\pm$ 5.3) than non-Saudis (8.4 $\pm$ 5.2) (*p*-value = 0.978) (Table 2).

On the other hand, participants from the southern (12.1 $\pm$ 6.0) and eastern (7.5 $\pm$ 5.9) regions scored the maximum and minimum average scores of anxiety, respectively indicating significant statistical differences. The pairwise comparison tests showed that participants from the southern region had scored significantly higher than those from the central region (*p*-value = 0.017) and the eastern region (*p*-value = 0.010) as well as participants from the western region had scored significantly higher anxiety than those from the eastern region (*p*-value = 0.047). In terms of educational levels, respondents with secondary or lower education (10.0 $\pm$ 5.2) and with diploma education (7.6 $\pm$ 4.6) had the maximum and minimum average scores of anxiety, respectively showing significant statistical differences (*p*-value = 0.002). The results of pairwise comparison tests showed that participants with secondary or lower educational levels had a significantly higher mean score of anxiety than those with diploma (*p*-value = 0.017) and university (*p*-value = 0.005) educational levels. Concerning work status, unemployed patients (9.1 $\pm$ 5.3) had scored higher than employed ones (7.7 $\pm$ 5.1) (*p*-value = 0.002). Regarding physical activity, physically inactive patients (10.1 $\pm$ 5.2) scored higher mean score of anxiety than those who are active (7.4 $\pm$ 4.9), showing a statistically significant difference (*p*-value < 0.001) (Table 2). The results also revealed that there was a statistically significant correlation between disease duration and depression ($\rho$ = 0.11, *p*-value = 016) meaning that patient with higher disease duration after diagnosis was associated with higher depression levels but this was not the case for anxiety ($\rho$ = 0.002, *p*-value = 0.967). Meanwhile, there was a negative and significant correlation between the time of the last MS relapse and anxiety levels ($\rho$ = – 0.15, *p*-value < 0.001). Furthermore, fatigue was positively correlated with anxiety, showing a statistically significant association ($\rho$ = 0.19, *p*-value < 0.001) (Table 3).

The results of the multiple linear regression are shown in Table 4. For reliability, the standard errors of the estimated coefficients and *p*-values are reported. The findings indicated that the estimated multiple linear regression model for depression was statistically significant (F = 7.40, *p*-value < 0.001), and the most important factors of depression were the patient’s sex, education, work status, physical activity, and fatigue. These factors had explained about 18.7% of the variability in the average depression scores reported by the participants. Specifically, the average depression score of males was 0.87 higher than their female counterparts ($\beta =$ 0.87; *p*-value = 0.033). Furthermore, the mean score of depression for participants with secondary or lower education was 1.34 higher than those with university education ($\beta =$ 1.34; *p*-value = 0.017) while this average for participants with diploma education was 1.35 less than those with university education ($\beta =$ – 1.35; *p*-value = 0.002), holding other things equal. Holding other variables constant, not employed patients were 1.22 times more depressed than employed ones ($\beta =$ 1.22; *p*-value = 0.002). Participants without physical activity were 1.79 more depressed than those with physical activity ($\beta =$ –1.79; *p*-value < 0.001), *citrus Barbus*. Moreover, one point increase in fatigue was associated with a 0.58 increase in the mean score of depression ($\beta =$ 0.58; *p*-value < 0.001), holding other things equal. On the other hand, age, marital status, region, nationality, last MS relapse, and duration of disease diagnosis had no simultaneous significant effect on depression (*p*-values > 0.05).

**Table 4:** Results of multiple linear regression for depression and anxiety

Variables	Categories	Depression		Anxiety	
		Coefficient (S.E)	*p*-value	Coefficient (S.E)	*p*-value
Age groups Ref. 18 – 29	30 – 39	– 0.10 (0.44)	0.826	–0.88 (0.57)	0.114
	40 -49	– 0.13 (0.58)	0.829	–0.79 (0.78)	0.279
	$\ge$ 50	0.75 (1.0)	0.470	–0.30 (1.45)	0.817
Sex Ref. female	Male	0.87^a^ (0.40)	0.033	0.68 (0.53)	0.184
Marital status Ref. married	Not married	0.17 (0.40)	0.670	0.34 (0.51)	0.492
Nationality Ref. Saudi	Non-Saudi	– 0.02 (0.52)	0.963	–0.19 (0.64)	0.772
Region Ref. central	Eastern	0.01 (0.54)	0.982	–0.86 (0.75)	0.205
	Western	0.91 (0.52)	0.078	1.95^a^ (0.61)	0.003
	Northern	– 0.91 (0.67)	0.176	–0.99 (0.75)	0.237
	Southern	-–0.45 (0.92)	0.625	2.05^a^ (1.19)	0.038
Education Ref. university	Secondary	1.34^a^ (0.48)	0.005	0.79 (0.59)	0.185
	Diploma	- 1.35^a^ (0.56)	0.017	–0.38 (0.61)	0.591
Work Status Ref. employed	Not employed	1.22^a^ (0.39)	0.002	1.32^a^ (0.49)	0.008
Physical activity Ref. No	Yes	–1.79^a^ (0.39)	< 0.001	–2.17^a^ (0.49)	< 0.001
Last MS relapse Ref. 3 – 6 months	7 – 12	– 0.37 (0.48)	0.447	–0.11 (0.61)	0.861
	13 – 18	– 0.31 (0.89)	0.729	0.59 (1.30)	0.593
	$>$ 18	– 0.84 (0.44)	0.059	–1.15^a^ (0.57)	0.040
Years after diagnosis of MS	-	0.04 (0.03)	0.245	-0.015 (0.04)	0.738
Fatigue	-	0.58^a^ (0.10)	< 0.001	0.45 (0.13)	< 0.001
Constant	-	0.46 (1.3)	0.729	3.38^a^ (1.67)	0.042

For depression model: R-squared 0.216; adjusted R-squared 0.187

For anxiety model: R-squared 0.165; adjusted R-squared 0.134

Robust standard errors in parentheses

^a^indicates significant effect at 5% level of significance

The results also indicated that the estimated multiple linear regression model for anxiety was statistically significant (F = 5.29, *p*-value < 0.001) and the included factors had explained about 13.4% of the variability in the average score of anxiety. Furthermore, the results revealed that factors of the region, work status, physical activity, last MS relapse, and fatigue were found to significantly affect the mean score of anxiety. That is, the average score of anxiety for patients from the western and southern regions were 1.95 and 2.05 times higher than those from the central region, respectively ($\beta =$ 1.95; 2.05; *p*-value = 0.003; 0.38), *ceteris paribus*. Unemployed patients were more anxious than those employed, holding other things constant ($\beta =$ 1.32; *p*-value = 0.008). Holding other variables constant, the average anxiety score of inactive patients was 2.27 times higher than those with physical activity ($\beta =$ –2.27; *p*-value < 0.001). Additionally, patients with more than 18 months since the last MS relapse were 1.15 times less anxious than those with 3 to 6 months since the last MS relapse ($\beta =$ –1.15; *p*-value = 0.040). With the *ceteris paribus* condition, a one-point increase in fatigue levels was associated with a 0.45 points rise in average anxiety score ($\beta =$ 0.45; *p*-value < 0.001). Nonetheless, none of the remaining variables had statistically significant impacts on patients’ anxiety (i.e., age groups, sex, marital status, nationality, educational levels, and disease duration) (Table 4).

## Discussion

The current study aimed at assessing factors influencing depression and anxiety levels in pwMS in KSA. The study revealed that the prevalence of depression and anxiety falls within the range of the findings of overseas research [7–11, 41, 42]. Meanwhile, participants were more likely to anxious rather than being depressed, which is consistent with some previous research [9, 10] but not others [8, 41, 42]. For instance, a study in Iran showed that severe anxiety was prevalent among 34.5% of patients and severe depression was prevalent in 24.1% of them as measured by the Depression, Anxiety, Stress Scale (DASS-21) [9]. Furthermore, Alsaadi et al. showed that the prevalence of anxiety and depression was 20% and 17.2%, respectively in 80 pwMS in the United Arab Emirates [10]. In Norway, however, the prevalence of depression (31.4%) was higher than anxiety prevalence (19.3%) among pwMS [41].

The univariate analysis showed that average scores of depression and anxiety were significantly different by educational levels, work status, physical activity with the highest prevalence being attached to those with secondary or lower education, without physical activity, and unemployed. Average scores of anxiety were significantly varied by region of residence with the highest prevalence in the southern region but differences in depression scores were not evident. Bivariate correlation showed that longer disease duration was associated with higher depression but not for anxiety. Moreover, having a recent relapse and high fatigue scores were associated with high anxiety and depression levels. The results of various studies are inconclusive. Karimi et al. showed that anxiety was inversely correlated with education confirming our findings but depression was not, which contradicted our study findings [9]. Furthermore, they showed that differences in mean scores of anxiety and depression were not significantly different by sex, age, and marital status, which is consistent with our findings [9]. On the other hand, Alsaadi et al. indicated that age, sex, marital status, education, and duration of MS had no significant correlation with both anxiety and depression [10]. A study by Greeke et al. showed that there is a highly significant correlation between depression and fatigue in pwMS [43].

The findings of the current study also revealed that factors including sex, education, work status, physical activity, and fatigue were significantly affecting depression levels, whilst region (e.g., central versus western and southern), physical activity, work status, last MS relapse (e.g, 3 -6 months versus > 18 months), and fatigue were significantly affecting anxiety levels in pwMS in KSA. A recent study in Norway indicated that the risk factors of depression were younger age at onset and fatigue, while those for anxiety were lower EDSS score, pain, fatigue, and younger age at onset [41]. Which is to some extent congruent with our findings. A study in Iran found that the education, occupation, and economic status of patients were all significant risk factors for depression, while the only significant factor affecting anxiety was the economic status [9]. Another study in Canada revealed that depression, low education, and decreased quality of life were associated with higher odds of anxiety [12]. Therefore, possible future intervention and educational programs are needed to support pwMS in KSA especially those with the lowest level of education. Disease duration was not an influential factor of both disorders, which is consistent with previous research [41, 42]. In this study, the average disease duration was 7.1 years which might be enough for pwMS to follow or develop appropriate coping strategies and adapt their lifestyle or quality of life [44]. In the meantime, this study showed that age, marital status, and nationality were not found to be determining factors of both disorders, which is mostly confirmed in previous research [9, 12, 13, 41, 42].

On the other hand, other research had considered other factors including genetic and pathophysiological pathways that develop the correlation between anxiety and depression and MS disease, which we do not anticipate in this study [45]. Nevertheless, since researchers were not able to link anxiety to MRI aberrations or clinical characteristics, some of them proposed that both disorders may be considered reactive responses to the onset and progression of MS disease [46]. However, a study by Bakshi *et al*. indicated that depression levels were significantly associated with total brain MRI lesions and atropy [47]. Therefore, premature treatment and intervention of anxious and depressive symptoms may contribute to reducing disease adverse outcomes.

The current study was subject to various limitations. First, the researcher relied on a self-report questionnaire for data collection that might have influenced results’ validity whereby participants in this study may not pay careful attention in completing the questionnaire, which may lead to a bias in measurement. Second, the current study studies anxiety and depression among pwMS who did not have walking difficulties, which might affect the generalizability of the results. In this regard, depression, anxiety, and fatigue will be probably higher if those patients were included. Third, MS disease type was not taken into account to classify patients. Therefore, classifying pwMS according to disease type may improve the results. Fourth, the design of the current study was cross-sectional, so results should be interpreted with caution since we are not able to determine a causal relationship between the variables. Finally, genetic, ecological, and vitamin D status are other factors that we did not control for in our study, which could have an impact on the rates of both disorders [48, 49].

Given the current COVID-19 pandemic, recent research had showed that pwMS were at higher risks of mental health disroders including depression, anxiety, stress, and insomnia due to the imposed lockdown that lead to distruption of lifestyle social distancing and isolation and thus urge the need for appropriate psychological support for this cohort [50, 51]. The current study, however, did not consider the COVID-19 related psychological impact in pwMS. Therefore, further research is needed to asses this effect, especially for patients who got infected with COVID-19 disease.

## Conclusions

In conclusion, the prevalence of anxiety was approximately twice the prevalence of depression in pwMS The current study showed that depression was more likely to be significantly affected by being male, of lower education, unemployed, inactive, and with a history of fatigue. While the anxiety was significantly affected by region, unemployment, the short period since last MS relapse, physical inactivity, and fatigue. Hence, increased focus on symptoms of anxiety and depression and their associated factors during regular clinic visits is a requirement. Finally, periodic tests and follow-up of treatment for pwMS suffering from both disorders by a psychologist are needed as well.

## Data Availability

The authors confirm that the data supporting the findings of this study are available within the article.

## References

[1] Heydarpour P, Khoshkish S, Abtahi S, Moradi-Lakeh M, Sahraian MA (2015). Multiple sclerosis epidemiology in Middle East and North Africa: a systematic review and meta-analysis. Neuroepidemiology.

[2] The Multiple Sclerosis International Federation Atlas of MS, 3rd ed, PART 1: Mapping multiple sclerosis around the world; Key epidemiology finding. 2020. https://www.atlasofms.org.

[3] AlJumah M, Bunyan R, Al Otaibi H, Al Towaijri G, Karim A, Al Malik Y, Kalakatawi M, Alrajeh S, Al Mejally M, Algahtani H, Almubarak A (2020). Rising prevalence of multiple sclerosis in Saudi Arabia, a descriptive study. BMC Neurol.

[4] Walton C, King R, Rechtman L, Kaye W, Leray E, Marrie RA, Robertson N, La Rocca N, Uitdehaag B, van der Mei I, Wallin M (2020). Rising prevalence of multiple sclerosis worldwide: Insights from the Atlas of MS. Mult Scler J.

[5] Koskie B. Multiple sclerosis by the numbers: facts, statistics, and you, Healthline. 2021. Available at: https://www.healthline.com/health/multiple-sclerosis/facts-statistics-infographic#Risk-factors.

[6] Dehghani R, KazemiMoghaddam V (2015). Potential causes of the in creased prevalence of multiple sclerosis in Iran: a review study. Pars J Med Sci..

[7] Nagaraj K, Taly AB, Gupta A, Prasad C, Christopher R (2013). Prevalence of fatigue in patients with multiple sclerosis and its effect on the quality of life. J Neurosci rural pract..

[8] Boeschoten RE, Braamse AM, Beekman AT, Cuijpers P, van Oppen P, Dekker J, Uitdehaag BM (2017). Prevalence of depression and anxiety in multiple sclerosis: a systematic review and meta-analysis. J Neurol Sci.

[9] Karimi S, Andayeshgar B, Khatony A (2020). Prevalence of anxiety, depression, and stress in patients with multiple sclerosis in Kermanshah-Iran: a cross-sectional study. BMC Psychiatry.

[10] Alsaadi T, El Hammasi K, Shahrour TM, Shakra M, Turkawi L, Mudhafar A, Diab L, Raoof M (2015). Prevalence of depression and anxiety among patients with multiple sclerosis attending the MS clinic at Sheikh Khalifa Medical City, UAE: cross-sectional study. Mult Scler Int.

[11] Wood B, Van Der Mei IA, Ponsonby AL, Pittas F, Quinn S, Dwyer T, Lucas RM, Taylor BV (2013). Prevalence and concurrence of anxiety, depression and fatigue over time in multiple sclerosis. Mult Scler J.

[12] Pham T, Jetté N, Bulloch AG, Burton JM, Wiebe S, Patten SB (2018). The prevalence of anxiety and associated factors in persons with multiple sclerosis. Multiple sclerosis and related disorders..

[13] Alsaadi T, El Hammasi K, Shahrour TM, Shakra M, Turkawi L, Almaskari B, Diab L, Raoof M (2015). Prevalence of depression and anxiety among patients with epilepsy attending the epilepsy clinic at Sheikh Khalifa Medical City, UAE: a cross-sectional study. Epilepsy Behav.

[14] Salehpoor G, Kafi S, Rezaei S, Hosseininezhad M, Salehi I (2014). Depression and sub-clinical markers of multiple sclerosis. Armaghan Danesh..

[15] Alhussain H, Aldayel AA, Alenazi A, Alowain F (2020). Multiple sclerosis patients in Saudi Arabia: prevalence of depression and its extent of severity. Cureus.

[16] Łabuz-Roszak B, Kubicka-Bączyk K, Pierzchała K, Machowska-Majchrzak A, Skrzypek M (2012). Fatigue and its association with sleep disorders, depressive symptoms and anxiety in patients with multiple sclerosis. Neurol Neurochir Pol.

[17] Rzepka M, Toś M, Boroń M, Gibas K, Krzystanek E (2020). Relationship between fatigue and physical activity in a polish cohort of multiple sclerosis patients. Medicina.

[18] Pokryszko-Dragan A, Dziadkowiak E, Zagrajek M, Slotwinski K, Gruszka E, Bilinska M, Podemski R (2016). Cognitive performance, fatigue and event-related potentials in patients with clinically isolated syndrome. Clin Neurol Neurosurg.

[19] Lerdal A, Celius EG, Moum T (2003). Fatigue and its association with sociodemographic variables among multiple sclerosis patients. Mult Scler J.

[20] Lakin L, Davis BE, Binns CC, Currie KM, Rensel MR (2021). Comprehensive approach to management of multiple sclerosis: addressing invisible symptoms—a narrative review. Neurology and therapy..

[21] D’Amico E, Zanghi A, Serra A, Murabito P, Zappia M, Patti F, Cocuzza S (2019). Management of dysphagia in multiple sclerosis: current best practice. Expert Rev Gastroenterol Hepatol.

[22] Calcagno P, Ruoppolo G, Grasso MG, De Vincentiis M, Paolucci S (2002). Dysphagia in multiple sclerosis–prevalence and prognostic factors. Acta Neurol Scand.

[23] Altmann P, Leutmezer F, Leithner K, Monschein T, Ponleitner M, Stattmann M, Rommer PS, Zrzavy T, Zulehner G, Berek K, Berger T. Predisposing Factors for Sexual Dysfunction in Multiple Sclerosis. Front Neurol. 2021:12. 10.3389/fneur.2021.618370 PMC7900565 33633671

[24] Guo ZN, He SY, Zhang HL, Wu J, Yang Y. Multiple sclerosis and sexual dysfunction. Asian J Androl. 2012;14(4):530–5. 10.1038/aja.2011.110 PMC3720075 22447199

[25] Alvino B, Arianna F, Assunta B, Antonio C, Emanuele DA, Giorgia M, Leonardo S, Daniele S, Renato D, Buscarinu MC, Massimiliano M (2021). Prevalence and predictors of bowel dysfunction in a large multiple sclerosis outpatient population: an Italian multicenter study. J Neurol.

[26] Browne C, Salmon N, Kehoe M (2015). Bladder dysfunction and quality of life for people with multiple sclerosis. Disabil Rehabil.

[27] Norton C, Chelvanayagam S (2010). Bowel problems and coping strategies in people with multiple sclerosis. B J Nurs.

[28] Dibley L, Coggrave M, McClurg D, Woodward S, Norton C (2017). “It’s just horrible”: a qualitative study of patients’ and carers’ experiences of bowel dysfunction in multiple sclerosis. J Neurol.

[29] Chiaravalloti ND, DeLuca J (2008). Cognitive impairment in multiple sclerosis. The Lancet Neurol..

[30] Sumowski JF, Benedict R, Enzinger C, Filippi M, Geurts JJ, Hamalainen P, Hulst H, Inglese M, Leavitt VM, Rocca MA, Rosti-Otajarvi EM (2018). Cognition in multiple sclerosis: State of the field and priorities for the future. Neurology.

[31] Trenova AG, Slavov GS, Manova MG, Aksentieva JB, Miteva LD, Stanilova SA (2016). Cognitive impairment in multiple sclerosis. Folia Med.

[32] Achiron A, Chapman J, Magalashvili D, Dolev M, Lavie M, Bercovich E, Polliack M, Doniger GM, Stern Y, Khilkevich O, Menascu S (2013). Modeling of cognitive impairment by disease duration in multiple sclerosis: a cross-sectional study. PloS one..

[33] Kahraman T, Özdoğar AT, Ertekin Ö, Özakbaş S (2019). Frequency, type, distribution of pain and related factors in persons with multiple sclerosis. Multi Scler Relat Disord..

[34] Mazhari A (2016). Multiple sclerosis-related pain syndromes: an imaging update. Curr Pain Headache Rep.

[35] Vickers MH (2009). Life and work with multiple sclerosis (MS): the role of unseen experiential phenomena on unreliable bodies and uncertain lives. Illn Crisis Loss.

[36] Zanghì A, Cimino S, Urzì D, Privitera S, Zagari F, Lanza G, Patti F, D’Amico E (2020). Pharmacotherapeutic management of lower urinary tract symptoms in Multiple Sclerosis patients. Expert Opin Pharmacother.

[37] Krupp LB, LaRocca NG, Muir-Nash J, Steinberg AD (1989). The fatigue severity scale: application to patients with multiple sclerosis and systemic lupus erythematosus. Arch Neurol.

[38] Al-Sobayel HI, Al-Hugail HA, AlSaif RM, Albawardi NM, Alnahdi AH, Daif AM, Al-Arfaj HF (2016). Validation of an Arabic version of fatigue severity scale. Saudi Med J.

[39] Zigmond AS, Snaith RP (1983). The hospital anxiety and depression scale. Acta Psychiatr Scand.

[40] Terkawi AS, Tsang S, AlKahtani GJ, Al-Mousa SH, Al Musaed S, AlZoraigi US, Alasfar EM, Doais KS, Abdulrahman A, Altirkawi KA (2017). Development and validation of Arabic version of the hospital anxiety and depression scale. Saudi J Anaesth.

[41] Beiske AG, Svensson E, Sandanger I, Czujko B, Pedersen ED, Aarseth JH, Myhr KM (2008). Depression and anxiety amongst multiple sclerosis patients. Eur J Neurol.

[42] Janssens AC, Van Doorn PA, De Boer JB, Van der Meche FG, Passchier J, Hintzen RQ (2003). Impact of recently diagnosed multiple sclerosis on quality of life, anxiety, depression and distress of patients and partners. Acta Neurol Scand.

[43] Greeke EE, Chua AS, Healy BC, Rintell DJ, Chitnis T, Glanz BI (2017). Depression and fatigue in patients with multiple sclerosis. J Neurol Sci.

[44] Lazarus RS, Folkman S (1984). Stress, Appraisal, and Coping.

[45] Haussleiter IS, Brüne M, Juckel G (2009). Psychopathology in multiple sclerosis: diagnosis, prevalence and treatment. Ther Adv Neurol Disord.

[46] Zorzon M, de Masi R, Nasuelli D, Ukmar M, Mucelli RP, Cazzato G, Bratina A, Zivadinov R (2001). Depression and anxiety in multiple sclerosis. A clinical and MRI study in 95 subjects. J Neurol.

[47] Bakshi R, Czarnecki D, Shaikh ZA, Priore RL, Janardhan V, Kaliszky Z, Kinkel PR (2000). Brain MRI lesions and atrophy are related to depression in multiple sclerosis. NeuroReport.

[48] Chwastiak L, Ehde DM, Gibbons LE, Sullivan M, Bowen JD, Kraft GH (2002). Depressive symptoms and severity of illness in multiple sclerosis: epidemiologic study of a large community sample. Am J Psychiatry.

[49] Zabad RK, Patten SB, Metz LM (2005). The association of depression with disease course in multiple sclerosis. Neurology.

[50] Zanghì A, D'Amico E, Luca M, Ciaorella M, Basile L, Patti F (2020). Mental health status of relapsing-remitting multiple sclerosis Italian patients returning to work soon after the easing of lockdown during COVID-19 pandemic: a monocentric experience. Mult Scler Relat Disord..

[51] Zarghami A, Hussain MA, Campbell JA, Ezegbe C, van der Mei I, Taylor BV, Claflin SB (2022). Psychological impacts of COVID-19 pandemic on individuals living with multiple sclerosis: a rapid systematic review. Mult Scler Relat Disord..

